# Increased susceptibility to *Mycobacterium avium* complex infection in miniature Schnauzer dogs caused by a codon deletion in *CARD9*

**DOI:** 10.1038/s41598-024-61054-x

**Published:** 2024-05-06

**Authors:** Keijiro Mizukami, Angella Dorsey-Oresto, Karthik Raj, Anna Eringis, Eva Furrow, Errolyn Martin, Daisuke Yamanaka, Alexandra Kehl, Ana Kolicheski, Vidhya Jagannathan, Tosso Leeb, Michail S. Lionakis, Urs Giger

**Affiliations:** 1 Section of Medical Genetics, School of Veterinary Medicine, University of Pennsylvania, Philadelphia, PA USA; 2 RIKEN Center for Integrative Medical Sciences, Laboratory for Genotyping Development, Yokohama, Kanagawa Japan; 3 Department of Veterinary Clinical Sciences, College of Veterinary Medicine, University of Minnesota, Saint Paul, MN USA; 4 Wildlife Center of North Georgia, Inc., Acworth, GA USA; 5 Fungal Pathogenesis Section, Laboratory of Clinical Immunology and Microbiology (LCIM), National Institute of Allergy and Infectious Diseases (NIAID), National Institutes of Health (NIH), Bethesda, MD USA; 6 Laboratory for Immunopharmacology of Microbial Products, School of Pharmacy, Tokyo University of Pharmacy and Life Sciences, Tokyo, Japan; 7 Laboklin GmbH & Co. KG, Bad Kissingen, Germany; 8 Department of Veterinary Pathobiology, College of Veterinary Medicine, University of Missouri, Columbia, MO USA; 9 Institute of Genetics, Vetsuisse Faculty, University of Bern, Bern, Switzerland; 10 Vetsuisse Faculty, University of Zürich, Zurich, Switzerland

**Keywords:** Primary immunodeficiency disorder, Zoonosis, Tuberculosis, Fungal infection, Hereditary defect, Canine, *Canis lupus familiaris*, Dog, Animal model, Genetics, Medical genetics, Animal breeding

## Abstract

Mammals are generally resistant to *Mycobacterium avium* complex (MAC) infections. We report here on a primary immunodeficiency disorder causing increased susceptibility to MAC infections in a canine breed. Adult Miniature Schnauzers developing progressive systemic MAC infections were related to a common founder, and pedigree analysis was consistent with an autosomal recessive trait. A genome-wide association study and homozygosity mapping using 8 infected, 9 non-infected relatives, and 160 control Miniature Schnauzers detected an associated region on chromosome 9. Whole genome sequencing of 2 MAC-infected dogs identified a codon deletion in the *CARD9* gene (c.493_495del; p.Lys165del). Genotyping of Miniature Schnauzers revealed the presence of this mutant *CARD9* allele worldwide, and all tested MAC-infected dogs were homozygous mutants. Peripheral blood mononuclear cells from a dog homozygous for the *CARD9* variant exhibited a dysfunctional CARD9 protein with impaired TNF-α production upon stimulation with the fungal polysaccharide β-glucan that activates the CARD9-coupled C-type lectin receptor, Dectin-1. While CARD9-deficient knockout mice are susceptible to experimental challenges by fungi and mycobacteria, Miniature Schnauzer dogs with systemic MAC susceptibility represent the first spontaneous animal model of CARD9 deficiency, which will help to further elucidate host defense mechanisms against mycobacteria and fungi and assess potential therapies for animals and humans.

## Introduction

The selective host resistance against specific viral, bacterial, fungal, or parasitic organisms observed in certain species is intriguing and, in some circumstances, explained by specific defense mechanisms^1^. Many genetic predispositions to infections have been described or are suspected in humans and companion animal species and are referred to as primary immunodeficiency disorders^1,2^. While certain genetic predispositions, such as severe combined immunodeficiency, cause broad-spectrum susceptibility to infection, other primary immunodeficiency disorders exhibit a narrower spectrum of infections, such as those caused by *Streptococcus,* non-tuberculous mycobacteria (NTM), and fungi^1,2^.

*Mycobacterium avium* complex (MAC) refers to intracellular NTM, including ubiquitous bacilli such as *M. avium* and *M. intracellulare* with several subspecies^3^. Furthermore, MAC has a wide range of animal reservoirs including wild and domestic mammals as well as birds^3,4^. However, mammals are mostly resistant to MAC infections (except ruminants with *M. avium* subsp. *paratuberculosis*), primarily due to potent IFN-γ/IL-12 protective responses that promote effective T cell-macrophage cross-talk and pathogen clearance^5^. While MAC infection is a zoonosis, its progression to clinical mycobacteriosis is primarily restricted to immunocompromised individuals, notably people with AIDS^6^ and, less commonly, to the young and elderly^7–9^.

Hereditary predisposition to mycobacterial infections related to variants in host defense genes have been suspected in people and animals for over a century^10^. Inborn errors in IFN-γ/IL-12-dependent immunity is associated with variants in many genes and has been reported to be associated with familial predisposition to mycobacterial disease in humans^5,11,12^. However, these defects account for only about half of the human patients with NTM infections^13^. Defects in the signaling pathway involving the C-type lectin receptor (CLR) signaling adaptor CARD9 underlie susceptibility to mucocutaneous and invasive candidiasis^14,15^ and other fungal infections in human patients^16–18^, and variants at the *CARD9* gene locus have also been identified in human patients with pulmonary NTM infections^19^.

Although dogs have considerable innate resistance to MAC infections^20^, severe systemic or disseminated MAC infections have been reported in the Miniature Schnauzer^4,21^, Basset Hound^22^, and sporadically in a few other dogs^23^. The present study aimed to unravel the mode of inheritance, molecular genetics, and possible immunological mechanisms related to the specific susceptibility to MAC infection in the Miniature Schnauzer breed.

## Methods

### Animals, ethics statement, and clinicopathological studies

All dogs studied were privately owned pets examined for illness or regular health screens with the consent of their owners, or samples were part of established/published canine DNA banks. The study samples were collected through the end of 2016 followed by worldwide genotyping surveys till 2023. This study was approved by the Institutional Animal Care and Use Committees of the University of Pennsylvania (A3079-01 and POAP #806003), Minnesota (#0908A70802, 1207A17243, and 1509-33019A), and Bern (Canton of Bern; BE 71/19). All studies were carried out in accordance with relevant guidelines and regulations, including ARRIVE and declaration of Helsinki. This study includes only dogs and clinical cases using small blood samples and cheek swabs submitted for diagnostics. There were no animal experiments, no experimental infections, no anesthesia, and no euthanasia of animals.

We gathered information on Miniature Schnauzers diagnosed with MAC infection from dog owners and breeders through breed club outreach, dog breeding forums, and global social media platforms. Reports from pet owners, breeders, and veterinary clinicians and signalment, clinical, and laboratory information were obtained from MAC-infected and related dogs when possible. Based upon the initial MAC-infected cases, a pedigree with confirmed MAC-infected and other related Miniature Schnauzer was compiled. Dogs were confirmed to be MAC-infected with bacterial culture and/or PCR for *M. avium* (all tested dogs had *M. avium* subsp*. hominissuis* infections) and/or histopathology/cytology showing macrophages containing numerous acid-fast red-staining rod-shaped bacilli through their respective routine clinical, pathology, and microbiology laboratories.

After the discovery of the putative pathogenic *CARD9* variant, data from a large genotyping survey of Miniature Schnauzers was conducted at the PennGen Laboratory of the University of Pennsylvania, Philadelphia, Pennsylvania, United States of America, as well as Laboklin, Bad Kissingen, Germany, and the data generated was included to determine the variant allele frequency in Miniature Schnauzers worldwide.

### Samples and DNA extraction

Genomic DNA was isolated from ethylenediaminetetraacetic acid (EDTA) blood, cheek swabs, and/or tissues, including liver and spleen, collected from infected and non-infected control Miniature Schnauzers and non-infected relatives of infected Miniature Schnauzers using QIAamp DNA Blood Mini kit and DNeasy Blood and Tissue kit (Qiagen, Valencia, CA). Once the putative pathogenetic variant was discovered, cheek swab samples from breeder- or pet-owned Miniature Schnauzers submitted for genotyping screening were analyzed.

### Genome-wide association study (GWAS) and homozygosity mapping

Genomic DNA from 8 MAC-infected and 160 non-infected Miniature Schnauzers was used in the GWAS analyses. The 160 non-infected dogs were all 5 years of age (twice the median age of MAC-infected dogs) or older with no history of MAC or other infections with opportunistic pathogens and were from a separate study on calcium oxalate urolithiasis based at the University of Minnesota. These dogs were recruited from Minnesota, Wisconsin, Iowa, North Dakota, and Illinois, and pedigrees were used to confirm that no first-degree relatives were included. Genotypes were generated using Illumina canine HD chips containing 173,686 evenly spaced single nucleotide variants (SNVs) in the dog CanFam3.1 genome sequence^24^. PLINK v1.07^25^ and genotyping results from the infected and control dogs were used to analyze the GWAS results. Individuals with marker call rates < 90% were removed from further analysis. Markers with individual call rates < 90%, markers with a minor allele frequency of < 5%, and markers strongly deviating from Hardy–Weinberg equilibrium (*p* < 1.0 × 10^–4^) were also excluded. PI-HAT values were used to confirm that none of the non-infected dogs were first-degree relatives (values < 0.5), and a multidimensional scaling (MDS) plot was generated to visually assess population structure. The Cochran-Mantel–Haenszel test was performed using the subpopulation information created by k-means clustering. The number of clusters was estimated by calculating within the group sum of squares. The influence of multiple testing was corrected by applying a Bonferroni correction to a *p* = 0.05 significance threshold. To further evaluate genome-wide significance, max(T) permutation testing of 100,000 permutations was applied. Genotyping data from the MAC-infected dogs and relatives was used to search for extended intervals of homozygosity in the area around the significant SNVs identified by GWAS compared to other genomic areas using Homozygosity Mapper^26^.

### Whole genome sequencing of MAC-infected dogs

Whole genome sequencing (WGS) was performed in 2 MAC-infected Miniature Schnauzers in 2015. PCR-free genomic libraries with an average insert size of 399–403 bp were prepared. The libraries were sequenced on an Illumina HiSeq 2500, and 421,755,598 and 325,571,202 paired-end reads (2 × 150 bp) corresponding to roughly 25- and 19-fold genome coverage were generated. Mapping to the CanFam3.1 dog reference genome assembly and variant calling of SNVs and small insertion or deletions variants (indels) was performed as described^27^. To filter for private variants in the 2 affected dogs, we used publicly available genome sequences from 660 control dogs of diverse breeds (Supplementary Table 1). Predictions of functional effects of the called variants were obtained with SnpEff software^28^ together with NCBI annotation release 105 on the CanFam 3.1 reference genome assembly.

### Direct DNA sequencing, genotyping, and screening for the discovered *CARD9* variant

The candidate variant, *CARD9*:XM_844178.5:c.493_495del, identified in MAC-infected Miniature Schnauzers was confirmed by direct Sanger sequencing of PCR products using genomic DNA, standard reagents, and procedures and the primer pair 5′-GACGCATCCGGGGAGTCAG-3′ and 5′-TCTGTGAGGAGACACCATCAGC-3′. A real-time PCR assay with allele-specific TaqMan minor groove binder probes (wild-type (WT): 5′-CTCCTCCTTGAGCCGC-3′, mutant: 5′-CACTCCTCGAGCCGC-3′) and dedicated primer pairs (5′-GGACAGCCTGCTTCGCA-3′, 5′-TCGTGGCTGCCAGCTT-3′) was developed to genotype the variant. Originally, a total of 274 Miniature Schnauzers were genotyped to confirm the phenotype-genotype correlation. Subsequent DNA screening for the pathogenic *CARD9* variant was offered and conducted through PennGen as well as Laboklin laboratories with support from the American and other Miniature Schnauzer Clubs worldwide.

### Immunological analyses

Heparin-anticoagulated whole blood was harvested from one clinically unaffected dog homozygous for the *CARD9* variant (*CARD9*^−/−^) and wild-type (*CARD9*^+/+^) dogs, and peripheral blood mononuclear cells (PBMCs) were isolated by density gradient centrifugation using lymphocyte separation media. To examine the integrity of CLR/CARD9 signaling in PBMCs, cells were stimulated with the fungal β-glucan OX-CA (100 μg/mL), as previously described^29^, and tumor necrosis factor-α (TNF-α) production was measured by ELISA at 48 h using canine TNF-α Quantikine ELISA Kit (R&D Systems, MN, USA). Furthermore, extracellular signal-regulated kinase (Erk) phosphorylation was determined by western blot analysis. For TNF-α analysis, PBMCs (5 × 10^5^ cells/well) were incubated in a round-bottom 96-well plate (BD Biosciences, CA, USA) with OX-CA (100 μg/mL) and cultured at 37 °C for 48 h in a humidified atmosphere of 5% CO_2_. Culture supernatants were stored at −80 °C until use. For analysis of Erk phosphorylation, PBMCs were stimulated with heat-killed *Candida albicans* (1 × 10^7^ cells/mL), OX-CA (100 μg/mL) or PMA (100 ng/mL), and ionomycin (1 μM) at 37 °C for 1 h, and Erk phosphorylation was determined by western blot analysis using primary antibodies against phospho-p44/42 (Phospho-Erk1/2, Thr202/Tyr204), p44/42 (Total-Erk1/2), as previously described^30^. Moreover, CARD9 expression was assessed in PBMCs with western blot analysis as previously described^29^. GraphPad Prism 8.0 (GraphPad Software, CA, USA) was applied for statistical analyses. The normality of the distribution of the data was determined by Shapiro–Wilk or Kolmogorov–Smirnov tests. Differences between data were then analyzed by two-tailed unpaired *t*-test (with or without Welch’s correction) or Mann Whitney *U*-test, where appropriate. *p* values < 0.05 were considered significant.

### Ethics declarations

This study was approved by the Institutional Animal Care and Use Committees of the University of Pennsylvania (A3079-01 and POAP #806003), Minnesota (#0908A70802, 1207A17243, and 1509-33019A) and University of Bern (Canton of Bern; BE 71/19).

## Results

### Historical, clinical, and pedigree information

Data from 23 MAC-infected Miniature Schnauzers showed the youngest was 1.5 years of age at diagnosis, the oldest was 8 years old, and the median age at diagnosis was 2.5 years. Sex distribution of MAC-infected Miniature Schnauzers was nearly equal (52% females), and affected Miniature Schnauzers had black/silver or salt/pepper coat colors. Evaluation of medical records of MAC-infected Miniature Schnauzers indicated that the most common initial clinical findings were lymphadenopathy (100%) followed by anorexia, lethargy, diarrhea, fever, hematochezia, hepatosplenomegaly, abdominal masses, lameness, and syncope. The extent of these findings seemed largely dependent on the infectious disease progression at the time of examination.

Laboratory test findings revealed mild non-regenerative anemia, mildly elevated serum liver enzyme activity values, and leukocytosis with few immature neutrophils. Necropsy findings included disseminated lymphadenopathy and widespread granulomatous infiltration of other tissues and bone marrow. Two MAC-infected dogs also had concurrent fungal skin infections (*Microsporum canis* or *Candida albicans*).

All MAC-infected Miniature Schnauzers for which information was available died or were euthanized within 1 year of diagnosis. Euthanasia was performed because of progressive serious illness, decline in quality of life, and concerns for zoonosis.

Data collection for 22 of the 23 MAC-infected Miniature Schnauzers revealed a common ancestor who was born in 1986 and produced 52 litters as recorded in the dog breed pedigree database, with its last reported litter born in 1998 (Supplementary Fig. 1). The common ancestor produced MAC-infected offspring when bred to its relatives. One affected dog did not have the dog as an ancestor in his pedigree papers, but parentage testing of the dog was not pursued. Offspring and close descendants of the common ancestor were exhibited internationally at dog shows and integrated into breeding programs in various countries. There is no genotypic and phenotypic information on the deceased common ancestor dog. The pedigree information is consistent with a simple autosomal recessive trait with a popular sire as the likely common ancestor. The actual founder may have been this popular sire or one of its ancestors.

### GWAS and homozygosity mapping

**Figure 1:**
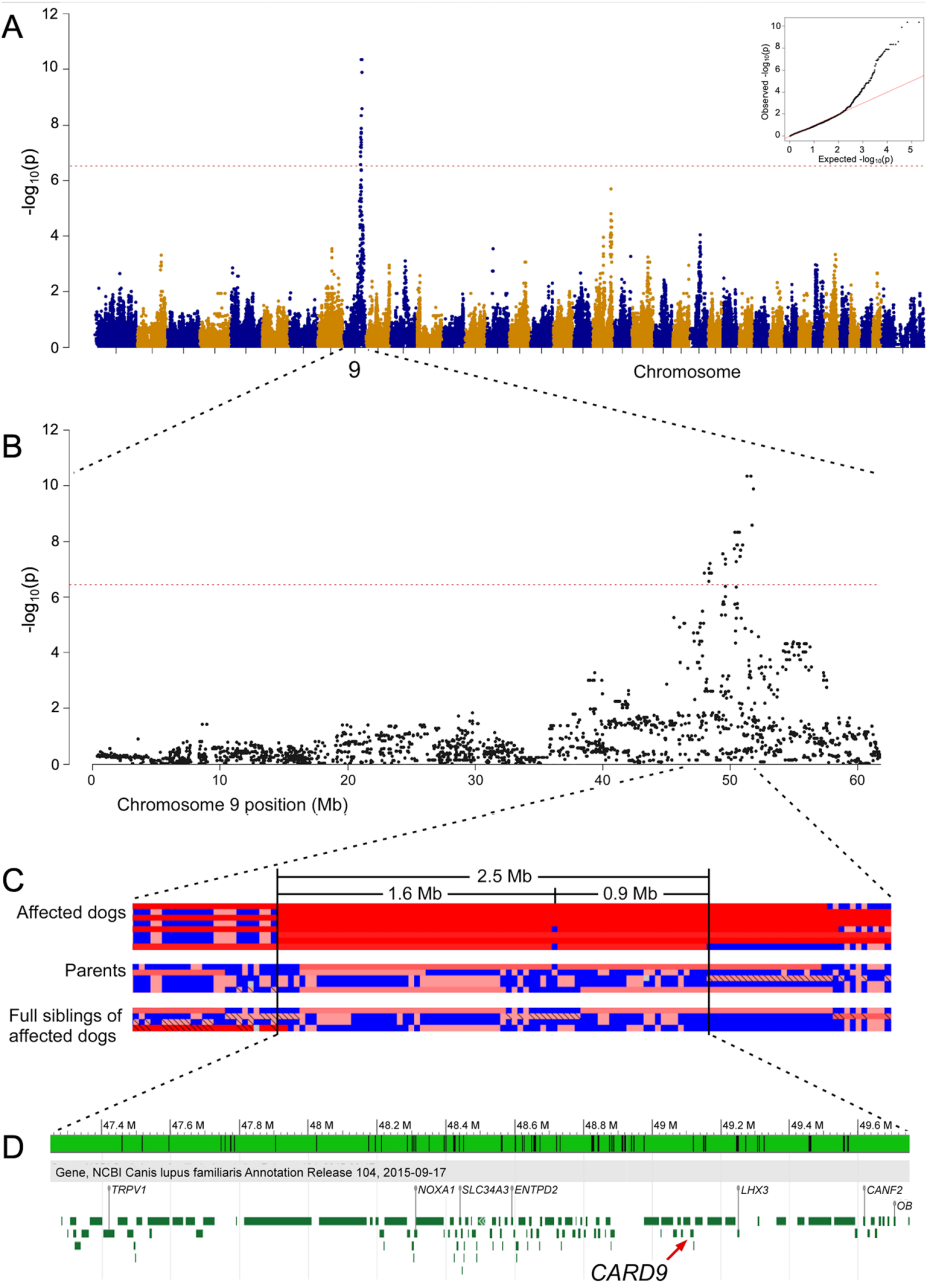
Genome-Wide Association Study mapping of the susceptibility to MAC infection in Miniature Schnauzers. (**A**) Manhattan plot from the association analysis using 8 MAC-infected Miniature Schnauzer cases and 160 controls indicates a signal with multiple associated SNVs on chromosome 9. The yellow line represents the Bonferroni-adjusted significance threshold of 0.05. (**B**) The detailed view of chromosome 9 suggests an associated interval of approximately 4 Mb at 50 Mb. (**C**) Homozygosity mapping. Each horizontal line corresponds to 1 of the 8 infected Miniature Schnauzers and 5 parents and 4 full siblings of the infected Miniature Schnauzers. The genotypes are indicated as colored boxes; blue represents heterozygous, grey unknown, and red homozygous genotypes. Longer homozygous stretches are drawn in a 'deeper' red than single homozygous markers. Genotypes homozygous for the minor allele are marked with a black diagonal bar. Infected dogs shared a total of 2.5 Mb consecutive homozygous segment. (**D**) NCBI genome viewer of the 2.5 Mb region in CamFam3. 118 genes including *CARD9* are annotated at the region.

Eight MAC-infected and 160 non-infected Miniature Schnauzers (> 5-year-old) were genotyped using Illumina canine_HD chips containing 173,686 evenly spaced markers. The raw data was pruned and a total of 74,446 markers were removed. Data from 2 control dogs were excluded as they failed quality control (< 90% call rate). Thus, the final GWAS dataset contained 99,240 SNVs from 8 MAC-infected not closely related and 158 non-infected Miniature Schnauzers. An MDS plot showed a skewed distribution of cases (Supplementary Fig. 2). To adjust for the population stratification due to cryptic relatedness and complex family structures often observed in purebred dogs^31^, the samples were divided into 3 clusters, and the clustering information was integrated into the subsequent association analyses. After the remaining stratification effect was adjusted by an inflation factor λ = 1.46, there were 32 significant markers (*p* < 5.04 × 10^–7^), all of which were located between 47.3 and 51.2 Mb on chromosome 9 in the CanFam3.1 assembly (Fig. 1A and 1B). The corrected *p*-value after permutation testing with 100,000 permutations was < 0.05 for 29 of the 32 markers.

Based on the shared common ancestor across MAC-infected Miniature Schnauzers, the infected individuals were expected to be identical by descent for the causative variant and flanking chromosomal segments. Homozygosity mapping revealed 2 adjacent extended regions of homozygosity (~ 1.5 and ~ 0.9 Mb: 47,295,065 to 49,732,677 bp) in all of the 8 MAC-infected dogs (Fig. 1C, Supplementary Table 2), but was not observed in the genomes of their 5 unaffected parents and 4 unaffected full siblings although one of the parents also had a run of homozygosity slightly shorter than 2.5 Mb at the region. There were 118 genes annotated in the ~ 2.5 Mb homozygous segment flanked by the heterozygous markers at positions 47,266,853 and 49,753,465, which we considered as a critical interval for the causal variant (Fig. 1D).

### Whole genome and direct DNA sequencing for the discovered *CARD9* variant, and in silico variant analyses

**Figure 2:**
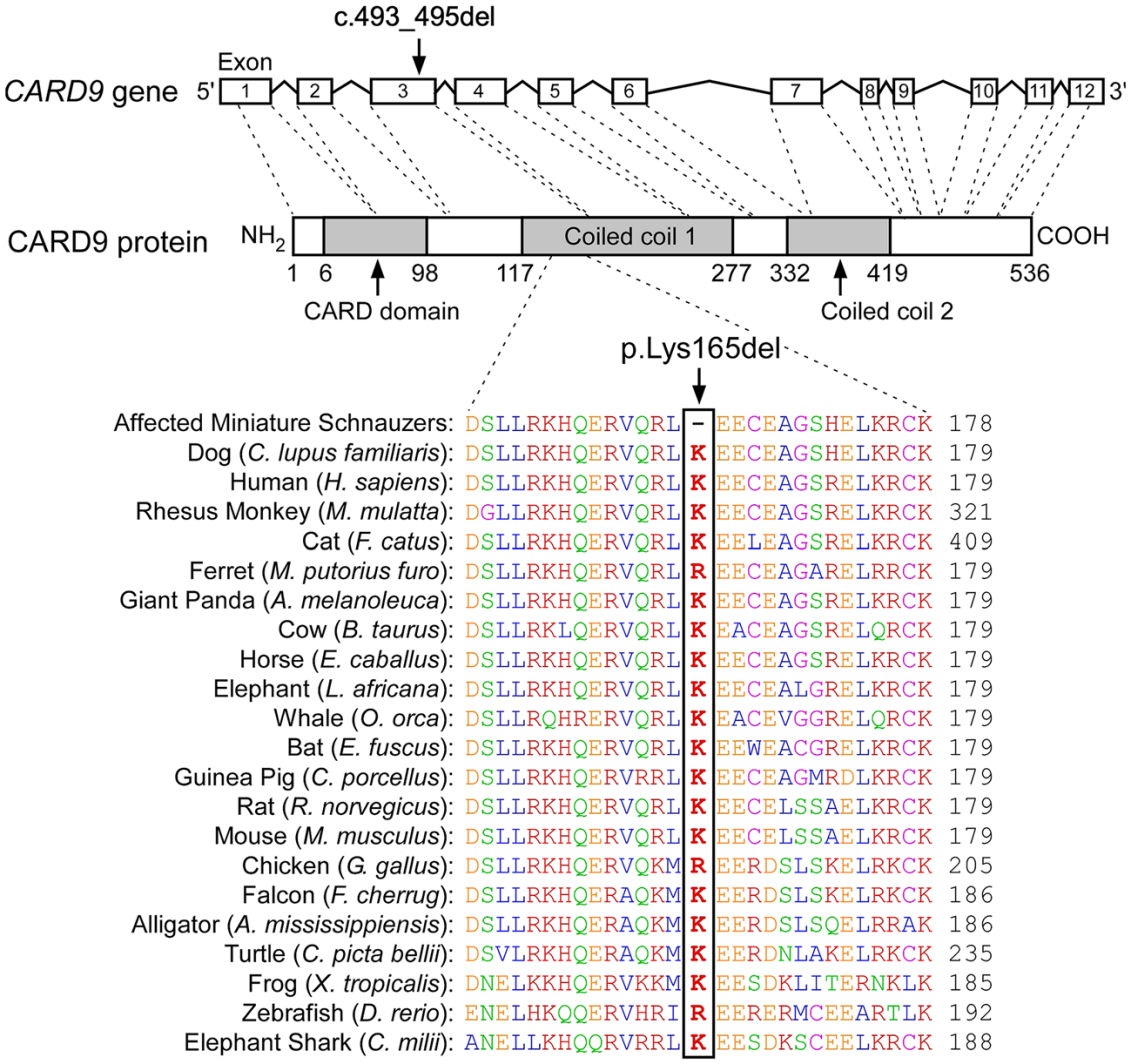
The single codon deletion in *CARD9* of MAC-infected Miniature Schnauzers. The canine *CARD9* gene is composed of 12 exons (ENSCAFT00000031299.1) and in MAC-infected Miniature Schnauzers a codon deletion exists in exon 3. The deleted amino acid is located in the first coiled-coil in CARD9 protein (E2R0V1-1), and the residue is highly conserved by lysine (K) or rarely arginine (R) in vertebrates.

The genomes of 2 sequenced MAC-infected Miniature Schnauzers were compared to data from control genome sequences derived from 660 dogs of diverse breeds. Across the entire genome, 2.3 million shared homozygous variants were detected in the 2 infected Miniature Schnauzers (Table 1). Only 20 of these variants were observed exclusively in the 2 MAC-infected Miniature Schnauzers and absent from all 660 control genomes. Of these 20 private variants, only one was located in the critical interval and appeared pathogenic (Supplementary Table 3): a 3 bp in-frame deletion in the *CARD9* gene, XM_844178.5: c.493_495del, predicted to result in the loss of a single lysine in the encoded protein, XP_849271.2: p.(165Lysdel). The variant was confirmed by direct DNA sequencing (Supplementary Fig. 3). Furthermore, PROVEAN v1.1.3^32^ predicted the deletion of ^165^Lys as deleterious with a score of −12.7 (a score < −2.5 is deleterious). The impacted amino acid residue in the CARD9 protein is conserved as lysine or rarely arginine in vertebrates and resides in a highly conserved coiled-coil motif of CARD9 (Fig. 2). According to 3 in silico coiled-coil structure prediction tools (CoCoNat, DeepCoil2, and CoCoPred)^33–35^, the variant was consistently predicted to disrupt the coiled-coil structure in CARD9 and thus be pathogenic (Supplementary Fig. 4).

**Table 1:** Results of homozygous variant filtering in 2 Miniature Schnauzers infected with *Mycobacterium avium* complex against 660 control genome sequences.

	Homozygous variants (SNVs and small indels)	
	In whole genome	In critical interval*
Shared variants in 2 affected dogs	2,270,064	5327
Private variants in 2 affected dogs	20	1
Private protein changing variants	1	1

SNV, single nucleotide variants. *Critical interval is Chr9:47,266,853–49,753,465 (CanFam 3.1).

### Initial genotyping for the discovered *CARD9* variant in the original study population

Genotyping for the candidate gene variant was initially performed in 274 Miniature Schnauzers, including 13 MAC-infected dogs, 58 relatives of the infected dogs, and 203 control Miniature Schnauzers from a separate study in the breed (Supplementary Table 4). This group included the 8 infected and 117 control dogs used in the GWAS. The genotyping results of these initial 274 Miniature Schnauzers were consistent with an autosomal recessive monogenic trait as predicted by pedigree analyses (Supplementary Fig. 1). All clinically MAC-infected dogs were homozygous for the mutant *CARD9* allele. Relatives of infected dogs were clinically asymptomatic and genotypically carriers (heterozygotes) or homozygous for the wild-type allele. All control dogs were homozygous for the wild-type allele.

### Screening for the discovered *CARD9* variant among Miniature Schnauzers

In a worldwide genotyping survey, DNA samples from a total of 7091 Miniature Schnauzers were collected (Supplementary Table 5). Overall, 4.78% and 0.14% of the Miniature Schnauzers were heterozygous and homozygous for the codon deletion in *CARD9*, respectively, while all other Miniature Schnauzers were homozygous for the wild-type allele. The samples from Miniature Schnauzers with one or both of the mutant *CARD9* alleles were from > 20 countries, including the USA, Canada, Argentina, South Africa, and countries in Europe. When pedigree information was available, they were all distantly related to a common ancestor, with a single exception. In fact, all heterozygous carriers analyzed by pedigrees had the same common ancestor born in 1986 in their pedigree (Supplementary Fig. 1). These genotyping survey results reveal a worldwide distribution of the mutant *CARD9* allele, and many of the identified homozygous MAC-infected and heterozygous Miniature Schnauzers were from Argentina^36,37^. All tested Miniature Schnauzers with MAC infection were homozygous for the *CARD9* variant. However, additional clinicopathological information for the MAC-infected and other Miniature Schnauzers that were genotyped was mostly lacking.

### Immunological functional analyses

**Figure 3:**
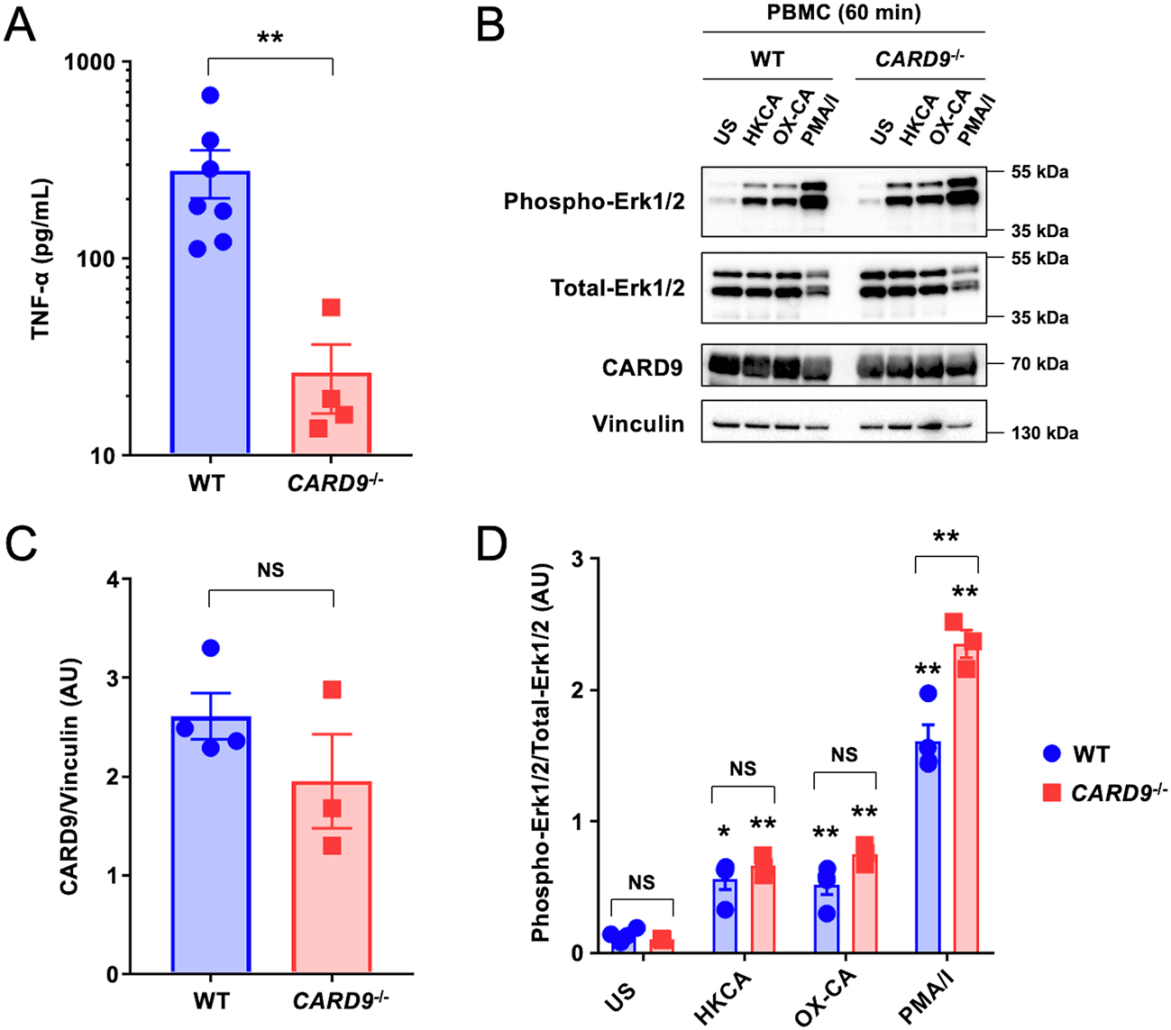
CARD9 expression and immunological functions of canine PBMCs with the homozygous c.Lys165del *CARD9* variant. (**A**) TNF-α production is decreased upon stimulation with OX-CA in PBMCs from dogs with the homozygous c.Lys165del *CARD9* variant (*CARD9*^−/−^) relative to wild-type (WT) dogs. n = 7 for WT and n = 4 for the studied dogs homozygous for the *CARD9* variant. **p < 0.01. (**B**–**D**) Representative cropped immunoblot images (**B**) and summary data of CARD9 (**C**) and pErk/Erk (**D**) in WT and *CARD9*^−/−^ canine PBMCs following stimulation with HKCA, OX-CA, or PMA/ionomycin. n = 4 for WT and n = 3 for *CARD9*^−/−^ dog PBMCs. Uncropped full-length images of the membrane are shown in Supplementary Fig. 5. Because these membranes were cut before hybridization with antibodies, the original membrane margins of these images are not available. All quantitative data represent mean ± SEM. At least 2 independent experiments were performed. *p < 0.05; **p < 0.01 when comparing unstimulated versus stimulated conditions and when comparing WT versus *CARD9*^−/−^ dog PBMCs for PMA/ionomycin, as indicated. NS, not significant; US, unstimulated; HKCA, heat-killed Candida albicans; OX-CA, NaClO-oxidized C. albicans; PMA/I, phorbol 12-myristate 13-acetate/ionomycin.

To understand the influence of the *CARD9* variant on the host innate immune response in dogs with and without the *CARD9* variant, canine PBMCs obtained from 1 healthy and 1 infected Miniature Schnauzer were stimulated with β-glucan (OX-CA), which induces CARD9-dependent cell activation via Dectin-1 engagement, and TNF-α production was evaluated^38^. Consistent with prior reports demonstrating that human and mouse CARD9 deficiency impairs the production of TNF-α by PBMCs upon β-glucan stimulation^29,38^, PBMCs obtained from the Miniature Schnauzer homozygous for the *CARD9* codon deletion exhibited significantly reduced (> 90%) TNF-α production upon OX-CA stimulation compared to the healthy dog (Fig. 3A).

While Erk phosphorylation following OX-CA stimulation is defective in PBMCs from human patients with *CARD9* variants that abrogate protein expression and in *CARD9*-null mouse cells^29,39–41^, Erk phosphorylation is maintained in human patients with *CARD9* missense variants encoding full-length dysfunctional protein^39,42^. PBMCs obtained from 1 Miniature Schnauzer homozygous for the *CARD9* codon deletion expressed a full-length CARD9 protein, as compared to wild-type canine PBMCs by immunoblot analysis (Fig. 3B–C, Supplementary Fig. 5). Concordantly, Erk phosphorylation was normal following stimulation with Dectin-1 agonists such as HKCA and OX-CA or with the CLR/CARD9-independent control stimulus PMA/ionomycin (Fig. 3C and D). These data collectively indicate that the *CARD9* codon deletion results in a full-length dysfunctional protein, which impairs TNF-α production but preserves Erk phosphorylation upon CLR engagement in canine PBMCs.

## Discussion

Through pedigree analyses, a GWAS, 2 WGS analyses, and a worldwide genotyping survey, we identified a unique codon deletion in the canine *CARD9* gene, p.(Lys165del), that is responsible for an autosomal recessive predisposition to systemic MAC infections with near complete penetrance in one breed of dogs (Miniature Schnauzers). This discovery confirms the involvement of the *CARD9* gene in host defense against MAC infections and provides the first naturally occurring animal model for the primary immunodeficiency disorder caused by CARD9 deficiency. According to variant interpretation guidelines, the identified *CARD9* variant in Miniature Schnauzers is classified as pathogenetic based upon the genotype to phenotype correlation, pedigree analyses, and genotypic survey as well as documented immunological impairments and computational analyses.

In humans and mice, CARD9 is composed of 536 amino acids with an N-terminal CARD domain and 2 coiled-coil domains downstream of the CARD domain (Uniport ID: Q9H257-1 in humans and A2AIV8-1 in mice). Based on the published genome sequence, the predicted canine CARD9 protein (XP_849271.2), also has 536 amino acids and has 87% and 84% homology to human and mouse CARD9, respectively. The 165th amino acid, deleted in all tested MAC-infected Miniature Schnauzers, is located in the first coiled-coil domain, which is highly conserved in vertebrates (Fig. 2).

Increased susceptibility to fungal infections has been associated with variants in *CARD9* in human patients (OMIM: 212050). Indeed, several *CARD9* coding changes in humans have been associated with infections with *Candida* (homozygous p.Gln295*)^14^, *Trichophyton* (homozygous p.Gln289* or p.Arg101Cys)^17^, *Exophiala* (homozygous p.Arg18Trp or p.Glu323*)^15^, *Phialophora* (compound heterozygous of p.Leu64fs*59 and p.Gln158* or homozygous p.Asp274fs*60)^43^ and *Aspergillus* (homozygous p.Met1Ile or p.Gln295*)^16^. The canine p.Lys165del that we identified corresponds to human p.Lys165del (CCDS48057.1 and CCDS6997.1) and, although it differs from the previously reported variants in CARD9-deficient human patients, it appears pathogenic based upon modeling programs. Miniature Schnauzers expressing this pathogenic *CARD9* variant had normal numbers of T-lymphocytes and a normal CD4:CD8 ratio, but they exhibited severely suppressed T-lymphocyte and moderately impaired B-lymphocyte responses in mitogen proliferation assays^44^, supportive of a primary immunodeficiency that likely contributes to loss of resistance to MAC.

CARD9 plays an important role in the ERK, p38, Jnk, and NF-κB signaling cascade induced by Dectin-1, Dectin-2, and NOD2 receptors and mononuclear and dendritic cells deficient in CARD-9 produce less IL-1β, IL-6, IL-8, IL-10, IL-12, IL-17, IFN-γ, and TNF-α when stimulated by fungi, intracellular bacteria, or the ligands of Dectin-1, Dectin-2, other CLRs, and NOD2 receptors^14,17,40,45–48^. Consistent with studies in human cells expressing *CARD9* missense variants encoding a full-length dysfunctional protein, we find that the *CARD9* codon deletion in Miniature Schnauzers results in a full-length dysfunctional protein, which impairs TNF-α production but preserves Erk phosphorylation upon CLR engagement in canine PBMCs. It should be noted these immunological studies were performed with a well-defined cell system for fungal infections and not specifically against mycobacteria. While inborn errors in IFN-γ/IL-12-dependent immunity are associated with familial predisposition to mycobacterial disease in humans^5,11,12^, the precise immune mechanisms regulating MAC clearance have not yet been elucidated. Bioengineered mice deficient in *CARD9* reveal that CARD9 is involved in the immune responses to *M. tuberculosis*^49^ and *Listeria monocytogenes*^45^, but these mice have not yet been experimentally challenged with MAC^45,50^. Furthermore, while unique heterozygous *CARD9* variants were identified in 6 human patients with pulmonary NTM infections^19^, whether they increased susceptibility to NTM infections was not determined. It should be noted that all *CARD9* variants previously reported in human patients with fungal infections were biallelic for nonsense or frameshift variants or deleterious missense variants according to PROVEAN prediction, and their heterozygous relatives did not show increased susceptibility to fungal or bacterial infections^14,15,17,43^.

Although the *CARD9* gene locus has been associated with non-infectious human diseases including inflammatory bowel disease^51^, ankylosing spondylitis^52^, IgA nephropathy^53^, and rheumatoid arthritis^54^, the clinical manifestations of *CARD9* defects have been primarily reported in patients with fungal diseases^14–17,43^. While all known Miniature Schnauzers homozygous for the *CARD9* variant developed MAC infections, a few in this study as well as in a separate case report^55^ also had evidence of fungal infections. One MAC-infected dog in this study exhibited generalized alopecia and was infected with *Microsporum canis*. Furthermore, fungal elements were identified in the myocardium of another Miniature Schnauzer with systemic MAC infection^55^, in which we confirmed homozygosity for the *CARD9* mutant allele. In another case report, *C. albicans* was detected in a lymph node of a Miniature Schnauzer infected with mycobacteria^56^, but samples from that dog were not available for genotyping to confirm the presence of the *CARD9* variant. While the discovered *CARD9* variant reported here may well be the cause of a broader spectrum of increased susceptibility to fungal, mycobacterial, and other intracellular infectious agents, further studies are needed.

The Miniature Schnauzer breed originated in Germany in the late nineteenth century and was officially recognized as a breed by the American Kennel Club in 1926^57^. The breed has become one of the popular breeds in the United States (17th of 184 AKC-recognized breeds in 2022)^58^ with active intercontinental breeding and use of popular dogs as sires. Our identification of the *CARD9* variant in MAC-susceptible Miniature Schnauzers and the associated finding that all MAC-infected Miniature Schnauzers and carriers for whom pedigree information was available were related to the same common ancestor from 1986, confirmed the opinion of breed clubs and breeders that a common ancestor and potentially its parents and offspring carried the genetic predisposition to MAC infection. As shown in the large *CARD9* genotyping survey over the past years, the mutant *CARD9* allele is distributed worldwide. While this suggested a high incidence of the *CARD9* variant, this is a biased survey because breeders of MAC-infected and carrier dogs are likely more interested in genotyping. Hopefully, continued genotyping and selective mating of clear to clear or clear to carrier dogs with subsequent genotyping in breeding programs will avoid the production of any dogs predisposed to MAC infections and reduce or eliminate the mutant *CARD9* allele in the breed.

MAC infections occur rarely in dogs except for predisposed Miniature Schnauzers and Basset Hounds^23^. MAC-infected Basset Hounds have the same clinical signs as seen in Miniature Schnauzers, and the ages of onset also range between 5 months and 5 years of age^22,59–61^. We sequenced the genome from 1 MAC-infected Basset Hound, but the dog did not show the *CARD9* variant found in Miniature Schnauzers or any other *CARD9* variants. Thus, identification of causal variants in distinct dog breeds will likely further inform us of the immune mechanisms responsible for MAC clearance.

In conclusion, this study identified a *CARD9* codon deletion present in the homozygous state in all Miniature Schnauzers infected with MAC, thereby documenting for the first time that CARD9 deficiency causes susceptibility to MAC infection in Miniature Schnauzer dogs. As worldwide dissemination of the mutant *CARD9* allele likely arose from the intensive use of a common and popular sire in the 1990s, screening for the *CARD9* variant is recommended within the breed to prevent the production of dogs at risk of developing MAC infection. While CARD9 deficiency is thought to cause increased susceptibility to fungal infection in human patients, only a few MAC-infected dogs with the *CARD9* variant identified in this study had concurrent fungal infections. Further characterization of dogs with CARD9 deficiency will offer new insight into host defense against MAC and fungal infections.

### Supplementary Information

Supplementary Figures.

Supplementary Information 1.

Supplementary Table 1.

Supplementary Table 2.

Supplementary Table 3.

Supplementary Table 4.

Supplementary Table 5.

## Data Availability

SNV genotypes for 168 dogs are given in File S1. Accession numbers of the 662 genome sequences are given in Supplementary Table 1.
