# Supplementary material for: Increased susceptibility to Mycobacterium avium complex infection in miniature Schnauzer dogs caused by a codon deletion in CARD9
Source: Sci Rep. 2024 May 6;14:10346. doi: 10.1038/s41598-024-61054-x (PMC11074286; doi:10.1038/s41598-024-61054-x)
Supplement: Supplementary file 1 — Supplementary Figures. [file 41598_2024_61054_MOESM1_ESM.pdf]

Increased Susceptibility to *Mycobacterium Avium* Complex Infection in Miniature Schnauzer Dogs Caused by a Codon Deletion in *CARD9*

Keijiro Mizukami,[1,2] Angella Dorsey-Oresto,[1] Karthik Raj,[1] Anna Eringis[1], Eva Furrow,[3] Errolyn Martin,[4] Daisuke Yamanaka,[5,6] Alexandra Kehl,[7] Ana Kolicheski,[8] Vidhya Jagannathan,[9] Tosso Leeb,[9] Michail S. Lionakis,[5] Urs Giger[1,10]

[1] Section of Medical Genetics, School of Veterinary Medicine, University of Pennsylvania, Philadelphia, Pennsylvania, United States of America

[2] Laboratory for Genotyping Development, RIKEN Center for Integrative Medical Sciences, Yokohama, Kanagawa, Japan

[3] Department of Veterinary Clinical Sciences, College of Veterinary Medicine, University of Minnesota, Saint Paul, Minnesota, United States of America

[4] Wildlife Center of North Georgia, Inc., Acworth, Georgia, United States of America

[5] Fungal Pathogenesis Section, Laboratory of Clinical Immunology & Microbiology (LCIM), National Institute of Allergy and Infectious Diseases (NIAID), National Institutes of Health (NIH), Bethesda, Maryland, United States of America

[6] Laboratory for Immunopharmacology of Microbial Products, School of Pharmacy, Tokyo University of Pharmacy and Life Sciences, Tokyo, Japan

[7] Laboklin GmbH & Co. KG, Bad Kissingen, Germany

[8] Department of Veterinary Pathobiology, College of Veterinary Medicine, University of Missouri, Columbia, MO, United States of America

[9] Institute of Genetics, Vetsuisse Faculty, University of Bern, Bern, Switzerland

[10] Vetsuisse Faculty, University of Zürich, Zürich, Switzerland

Corresponding authors: Keijiro Mizukami keijiro.mizukami@riken.jp and Urs Giger giger@upenn.edu

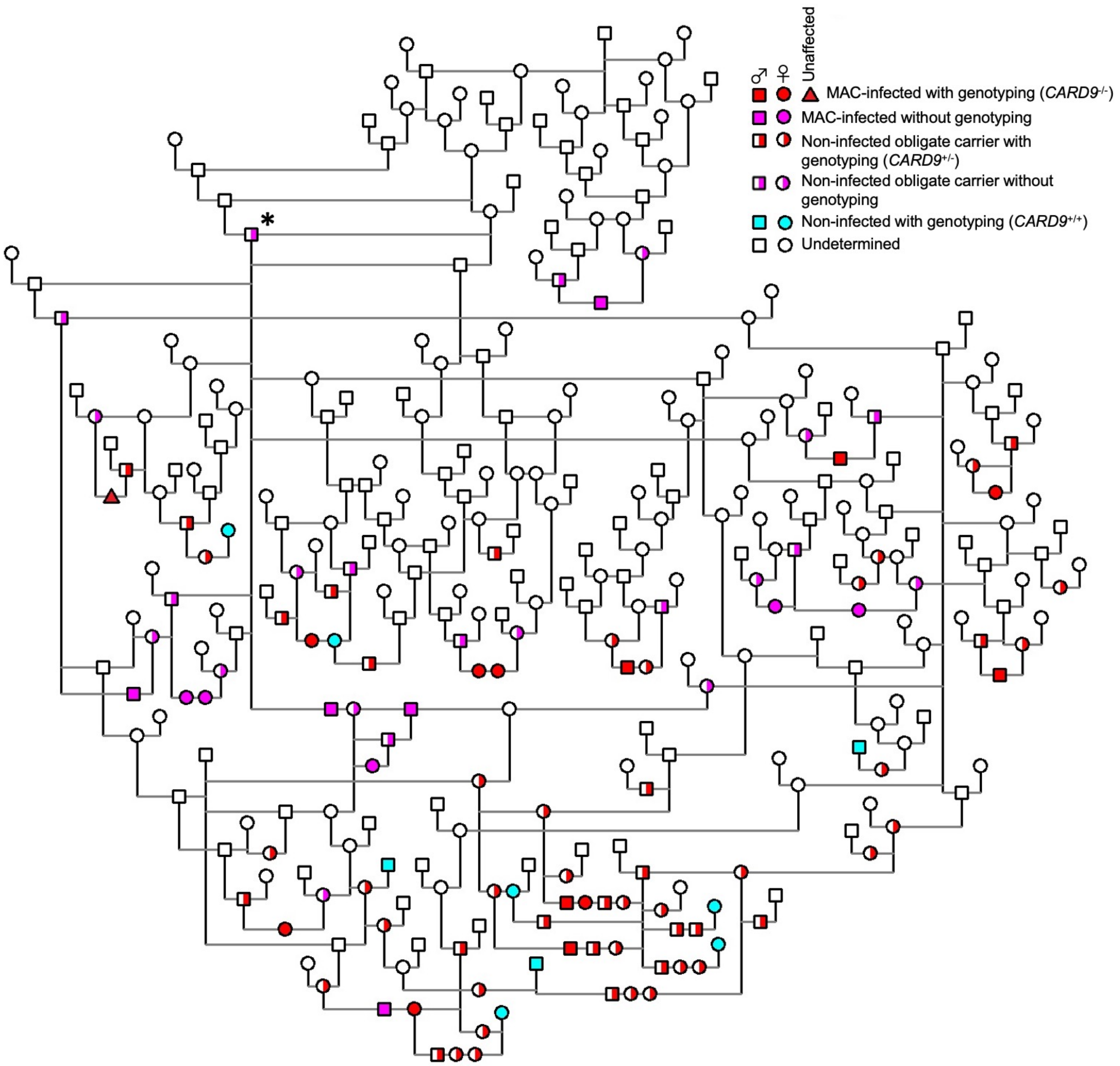

Supplementary Figure 1. Pedigree analysis of Miniature Schnauzer family with MAC infection. All infected Miniature Schnauzers and carriers shared a common male Miniature Schnauzer born in 1986 except for one affected dog whose parentage was not confirmed (*).

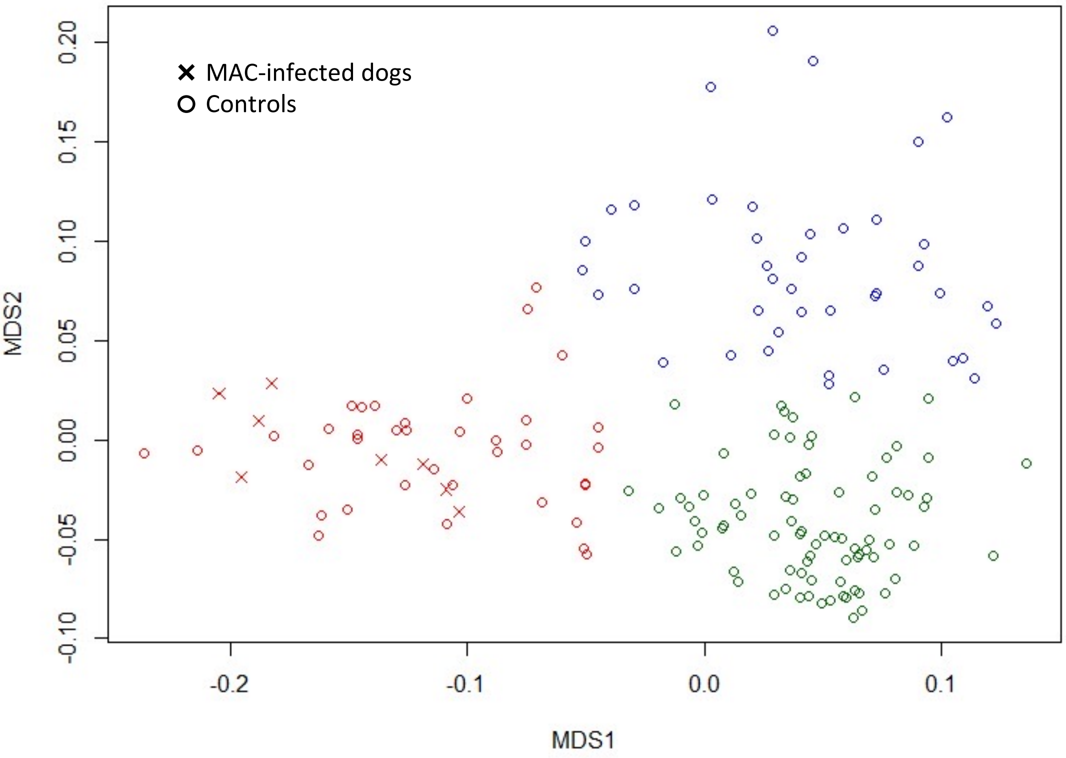

Supplementary Figure 2. Multidimensional scaling plot of MAC-infected dogs and controls. Subpopulations created by k-means clustering (K = 3) are indicated by color (red, blue, and green).

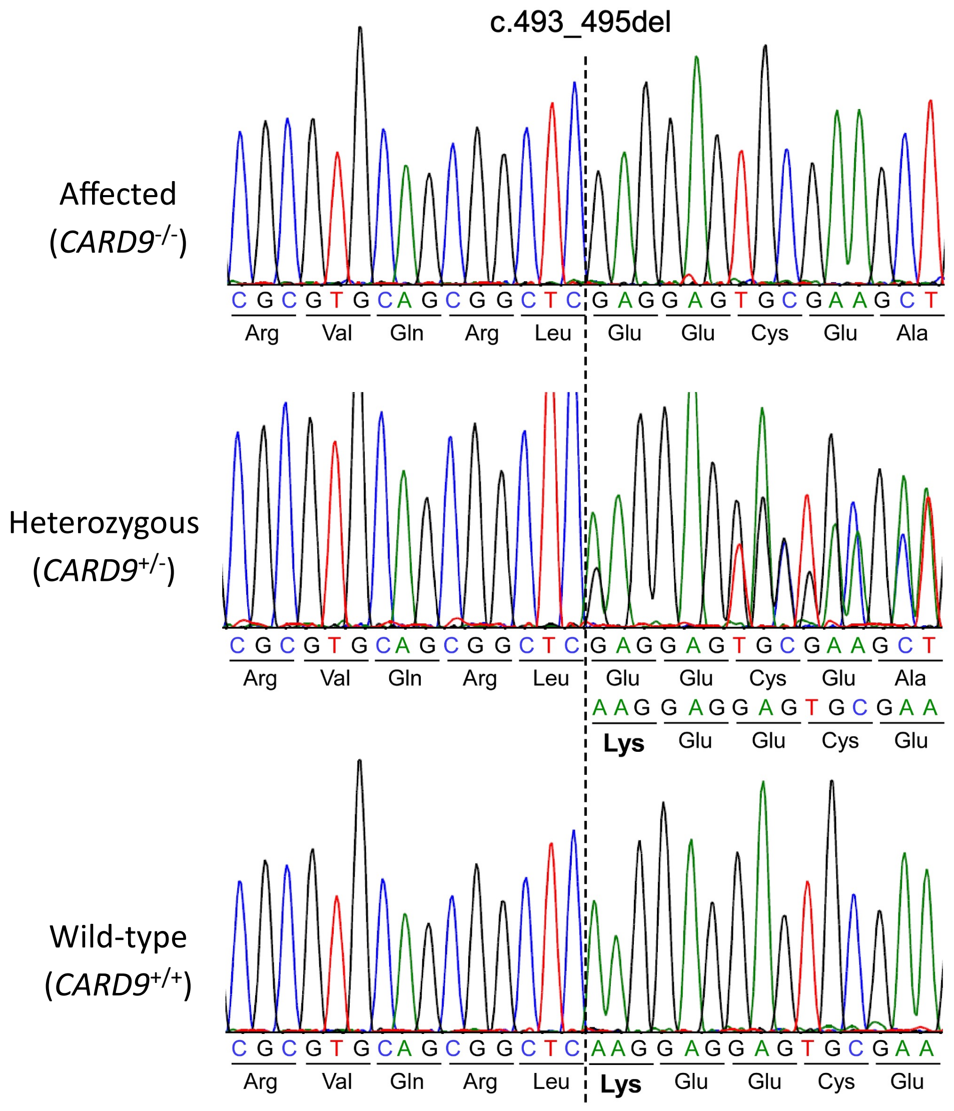

Supplementary Figure 3. Genomic DNA sequencing chromatograms around the 3 bp deletion in the *CARD9* gene. Infected dogs were homozygous for the deletion, while the one parent and control dog were either heterozygous or wild-type homozygous, respectively. These studies confirm an autosomal recessive trait of inheritance.

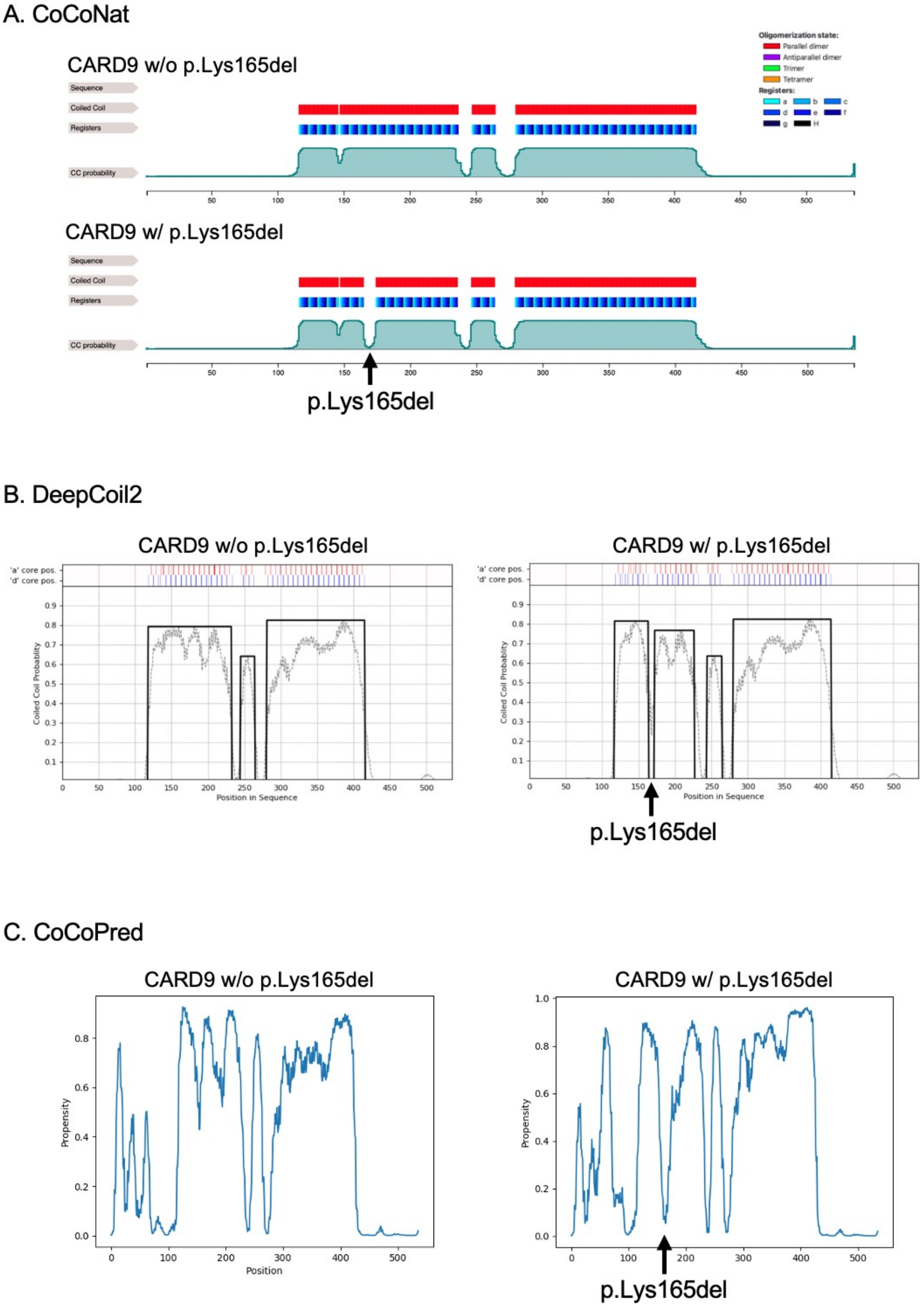

Supplementary Figure 4. Results of *in silico* coiled-coil structure prediction of CARD9 with wild-type sequence and with c.Lys165del variant. Prediction was conducted using 3 tools: CoCoNat (A), DeepCoil2 (B), and CoCoPred (C).

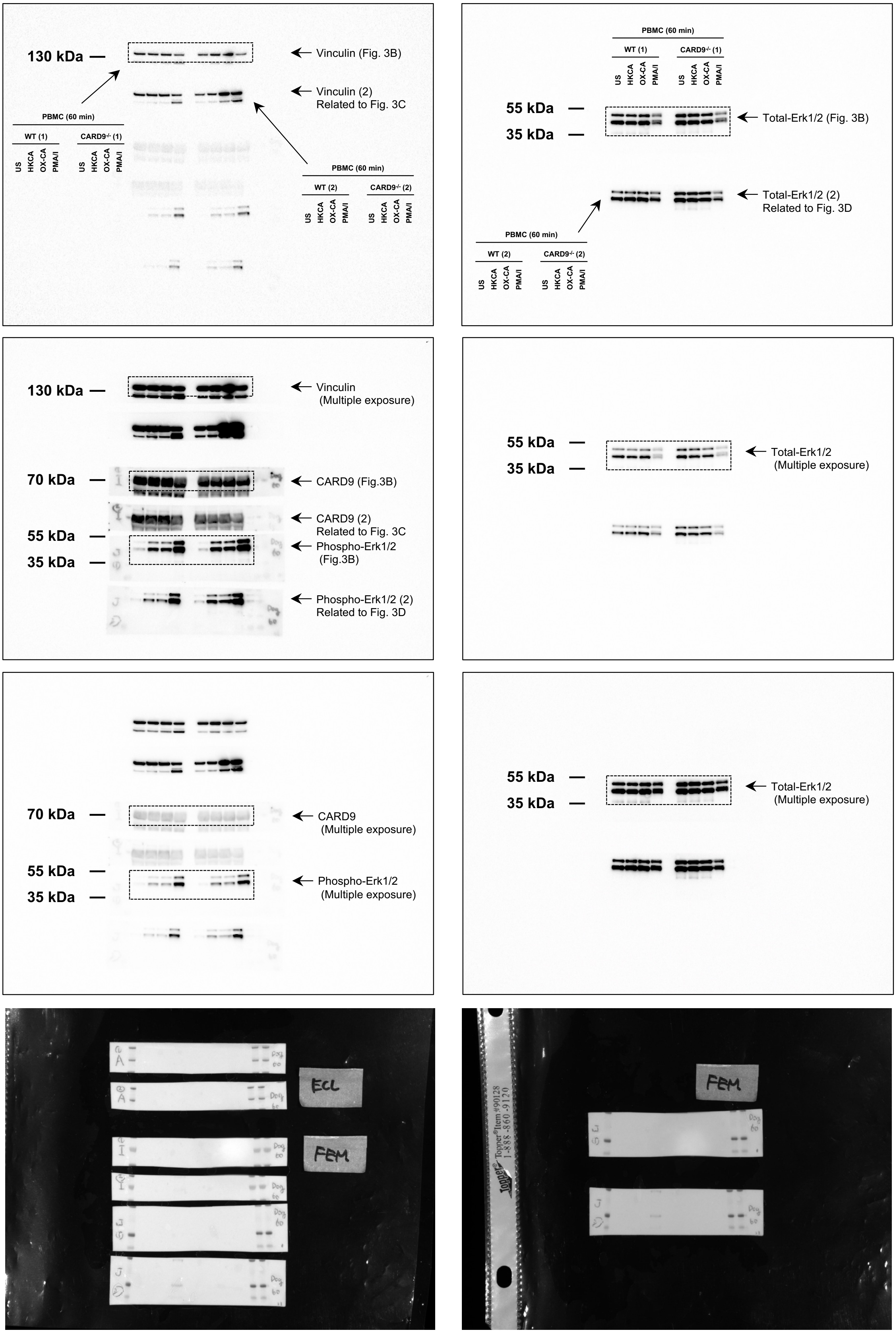

Supplementary Figure 5. Near full-length immunoblot images of Figure 3B. The cropped parts shown in Figure 3B were indicated by a square dotted line in this supplemental figure. Multiple exposure images were also included. Because these membranes were cut before hybridization with antibodies, the original membrane margins of these images are not available.

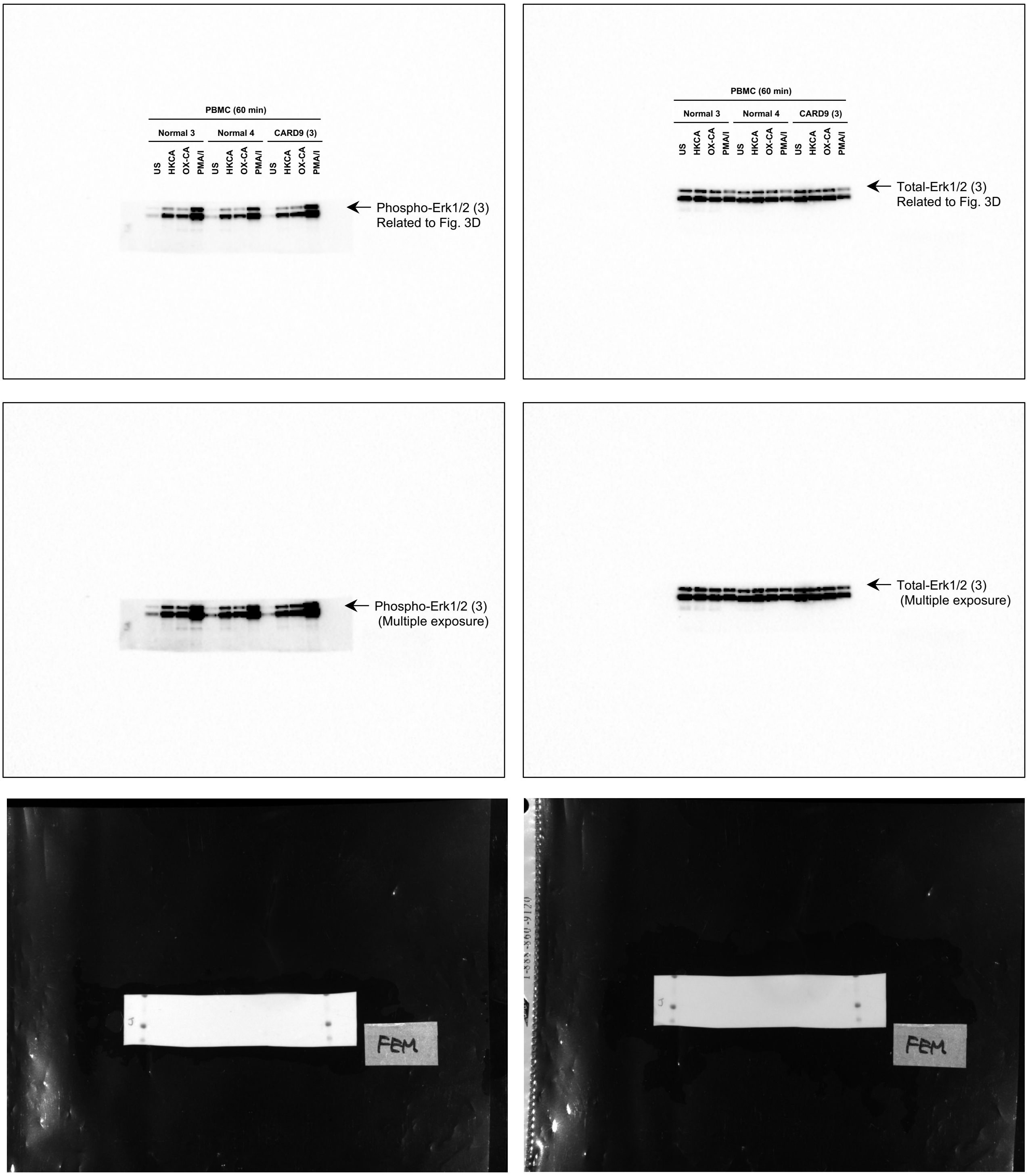

Supplementary Figure 5 (continued). Near full-length immunoblot images of Figure 3B. The cropped parts shown in Figure 3B were indicated by a square dotted line in this supplemental figure. Multiple exposure images were also included. Because these membranes were cut before hybridization with antibodies, the original membrane margins of these images are not available.

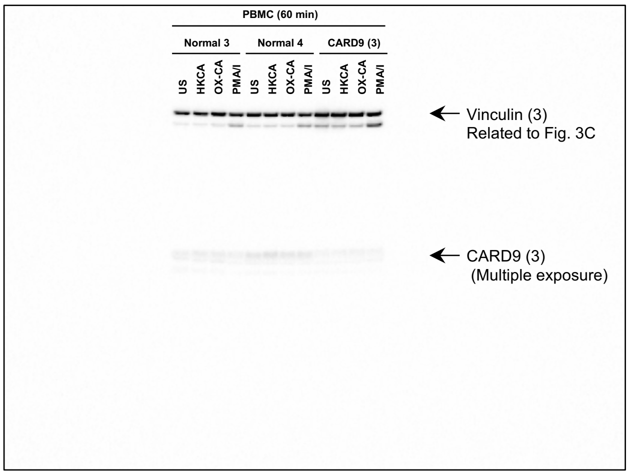

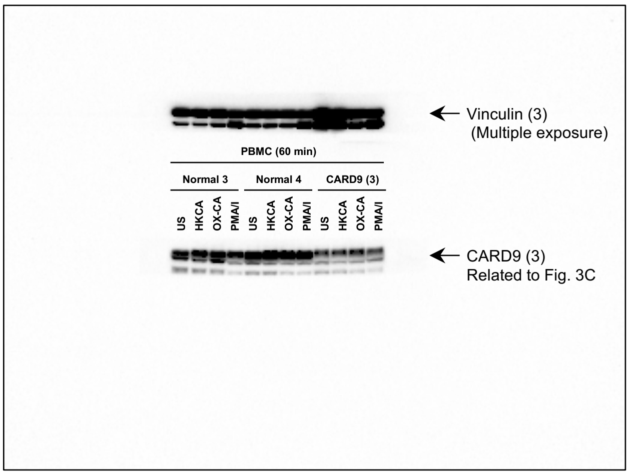

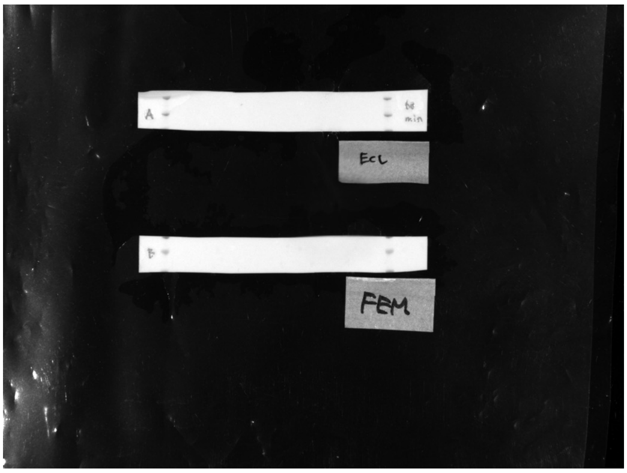

Supplementary Figure 5 (continued). Near full-length immunoblot images of Figure 3B. The cropped parts shown in Figure 3B were indicated by a square dotted line in this supplemental figure. Multiple exposure images were also included. Because these membranes were cut before hybridization with antibodies, the original membrane margins of these images are not available.
